# Clinicopathological and Prognostic Value of Necroptosis-Associated lncRNA Model in Patients with Kidney Renal Clear Cell Carcinoma

**DOI:** 10.1155/2022/5204831

**Published:** 2022-05-23

**Authors:** Jun Gu, Zexi He, Yinglong Huang, Ting Luan, Zhenjie Chen, Jiansong Wang, Mingxia Ding

**Affiliations:** Department of Urology, The Second Affiliated Hospital of Kunming Medical University, Yunnan Institute of Urology, Kunming, 650000 Yunnan Province, China

## Abstract

**Background:**

Necroptosis, a recently identified type of programmed necrotic cell death, is closely related to the tumorigenesis and development of cancer. However, it remains unclear whether necroptosis-associated long noncoding RNAs (lncRNAs) can be used to predict the prognosis of kidney renal clear cell carcinoma (KIRC). This work was designed to probe the possible prognostic worth of necroptosis-associated lncRNAs along with their impact on the tumor microenvironment (TME) in KIRC.

**Methods:**

The Cancer Genome Atlas (TCGA) database was used to extract KIRC gene expression and clinicopathological data. Pearson correlation analysis was used to evaluate necroptosis-associated lncRNAs against 159 known necroptosis-associated genes. To define molecular subtypes, researchers used univariate Cox regression analysis and consensus clustering, as well as clinical significance, TME, and tumor immune cells in each molecular subtype. We develop the necroptosis-associated lncRNA prognostic model using univariate Cox regression analysis and least absolute shrinkage and selection operator (LASSO) regression analysis. Patients were divided into high- and low-risk groups according to prognostic model. Moreover, comprehensive analyses, including prognostic value, gene set enrichment analysis (GSEA), immune infiltration, and immune checkpoint gene expression, were performed between the two risk groups. Finally, anticancer drug sensitivity analyses were employed for assessing associations for necroptosis-associated lncRNA expression profile and anticancer drug chemosensitivity.

**Results:**

Through univariate analysis, sixty-nine necroptosis-associated lncRNAs were found to have a significant relationship with KIRC prognosis. Two molecular clusters were identified, and significant differences were found with respect to clinicopathological features and prognosis. The segregation of patients into two risk groups was done by the constructed necroptosis-associated lncRNA model. The survival prognosis, clinical features, degree of immune cell infiltration, and expression of immune checkpoint genes of high-risk and low-risk groups were all shown to vary.

**Conclusions:**

Our study identified a model of necroptosis-associated lncRNA signature and revealed its prognostic role in KIRC. It is expected to provide a reference for the screening of KIRC prognostic markers and the evaluation of immune response.

## 1. Introduction

Renal cell carcinoma (RCC) represents a highly prevalent global malignancy within the genitourinary tract and ranks second, after prostate and bladder cancer [1]. In 2020, there were over 430,000 newly diagnosed cases, resulting in nearly 180,000 deaths [2]. Kidney renal clear cell carcinoma (KIRC) is the predominant RCC histology-subtype, forming approximately 70% of all RCC cases [3]. Cigarette smoking, obesity, and hypertension are high-risk parameters for RCC, although their relative effects can vary across populations [4]. Notwithstanding a plethora of therapeutic option availability, including surgery, radiotherapy, chemotherapy, and targeted drug therapy, partial/radical nephrectomy remains the ideal therapeutic course adopted against early stage or localized KIRC with definite efficacy. In addition, local recurrence or distant organ metastasis still occurs in 20% ~40% of KIRC patients postsurgery [5]. Currently, there are several targeted drugs accepted for first-/second-line therapy against metastatic RCC, including sorafenib [6], sunitinib [7], pazopanib [8], axitinib [9], lenvatinib [10], and cabozantinib [11]. However, multiple patients show distinct treatment responses to targeted drug therapy, which could be related to intratumor and intertumor heterogeneity [12, 13]. Such restrictions lead to reduced prognostic odds, aggravated through a lack of KIRC-predictive biomarker repertoires, consequently requiring the generation of such a novel prognostic modelling system.

Apoptosis is the main form of cellular mortality within living organisms. In recent years, through extensive research on the mechanism of cell death, emerging methods of cell death have been discovered and described. Degterev et al. [14] first reported and named a new cell death mode—necroptosis. Necroptosis shares a common pathway with apoptosis [15, 16]. TNF-*α* combines with TNFR1-linked death domain protein, Fas-associated death domain, pro-caspase-8, and RIPK1 to form complex IIa, which promotes caspase 8 activation. Activated caspase 8 induces apoptosis by activating caspase 3 [17]. Whenever caspase 8 activity is downregulated, RIPK1, RIPK3, and MLKL form complex IIb, also termed necrosome [16]. Phosphorylated MLKL causes plasma membrane rupture, consequently inducing necroptosis [18, 19]. Similarly, the morphological characteristics of necroptosis are degradation of lysosomal membrane, vacuolation of cytoplasm, disintegration of plasma membrane, and cell rupture. Following further research, necroptosis is confirmed as having an important position within multiple cancer models, including renal [20], colorectal [21], ovarian [22], and breast cancer [23]. Furthermore, latest investigations have shown that antiapoptotic tumor cells may be sensitive to the necrosis pathway [24, 25], suggesting that it is necessary to search for additional biomarkers involved in necroptosis to understand the interplays concerning KIRC necroptosis and have appropriate prognostic predictive capacities for KIRC cases.

Meanwhile, long noncoding RNAs (lncRNAs) refer to RNA molecules spanning over 200 nt long with nil protein-coding potential. This was once considered part of the “dark matter” of the genome with no biological functions being assumed [26]. Presently, a plethora of lncRNAs have been identified, and several lncRNAs are now believed to function as important regulatory molecules [27]. Current investigations identified that lncRNAs can be implicated within multiple processes, including epigenetic remodeling [28], regulating chromatin structure [29], regulating RNA stability [30], and acting as microRNA sponges [31]. Presently, literature is scarce regarding necroptosis-associated lncRNAs. In a related investigation, Harari-Steinfeld et al. [32] highlighted that lncRNA H19-derived miR-675 promoted human hepatocellular carcinoma cell necroptosis in response to inflammation symptoms by targeting Fas-linked protein with death domain. One recent study revealed that the tumor suppressor p53 upregulates lncRNA TRINGS in a direct manner during glucose starvation conditions. The lncRNA TRINGS interacts with STRAP and thwarts STRAP-GSK3*β*-NF-*κ*B necrotic pathways [33]. Moreover, other studies have found that myocardial ischemia-reperfusion injury can lead to abnormal expression of lncRNAs in cardiomyocytes, thus affecting various cellular functions such as mitochondrial homeostasis, apoptosis, necrosis, and autophagy [34]. However, functions adopted by necroptosis-associated lncRNAs for treating or prognostic determination of KIRC are still unclear. This investigation focused on identifying necroptosis-associated lncRNAs within KIRC and developed a prognosis-associated lncRNA model of dysregulated necroptosis-associated lncRNAs. We then explored functions adopted by necroptosis-associated lncRNAs within the immune microenvironment, prognostic odds determination, and anticancer drug sensitivity of KIRC.

## 2. Materials and Methods

### 2.1. Data Acquisition

Transcriptomic data and clinical information of 539 KIRC cases were obtained through TCGA database (http://cancergenome.nih.gov/). Cases having comprehensive clinical/pathology/prognostic odds data were enrolled in this investigation for additional research. Thirteen patients with unavailable overall survival (OS) and clinical information were removed from the investigation. Overall, 526 cases having matching tumor tissue/medical data were enrolled within this investigation.

### 2.2. Identifying Necroptosis-Associated lncRNAs

The Kyoto Encyclopedia of Genes and Genomes (KEGG) pathway (https://www.kegg.jp/entry/hsa04217) was utilized for locating 159 genes tied with necroptosis. Protein-coding genes and lncRNAs were annotated through Ensembl human genome browser GRCh38.p13 (http://asia.ensembl.org/index.html). Necroptosis-associated lncRNAs were recognized through Pearson correlation analysis (|*R*| > 0.7 and *p* < 0.001). This study probed differentially expressed necroptosis-associated lncRNAs between cancer and normal samples using the limma package for statistical significance. The cutoff value was |log2FC| > 1 and FDR < 0.05 (fold change (FC); false discovery rate (FDR)). Univariate Cox regression analysis was employed for constructing prognosis-/necroptosis-associated lncRNA expression profiles through the R “survival” package [35] (*p* < 0.05).

### 2.3. Determination of Prognostic Molecular Player Subtypes

In order to investigate potential roles adopted by necroptosis-associated lncRNAs within KIRC, “ConsensusClusterPlus” package [36] in R was employed for recognizing crystallized molecule-based nexa, depending upon necroptosis-associated lncRNA expression profiles collected through univariate Cox regression analysis. Using repeated sampling procedure, 80% of all cases were assayed 1,000 times. Similarity distances across cases were determined through Euclidean distance, with *K*-means employed in clustering. The criteria for defining cluster quantities were low coefficient of variation, high consistency within clusters, and no significant increase within the area under the curve (AUC) of the cumulative distribution function (CDF).

### 2.4. Molecular-Based Subtyping Clinical Importance

In order to study the medical importance for two specific KIRC molecular-based subtypes, associations across molecular-based subtypes, clinical features, and prognostic odds were examined. Collected clinical features of KIRC cases included age, gender, TNM stage, and tumor-grade/-stage. Consequently, the investigation focused on OS variations across two differing KIRC case clusters using Kaplan–Meier method. Meanwhile, this was presented through “survival” and “survminer” packages in R [35].

### 2.5. Immune-System Infiltration Study for Molecular Subtypes

The tumor microenvironment (TME) scores could predict efficacy of immunotherapy well. The immune score, interstitial score, and tumor purity were employed for evaluating TME scores across differing clusters, measuring through “estimate” R package [37]. Cell-type Identification By Estimating Relative Subsets Of RNA Transcripts (CIBERSORT) was consistently proven to be reliable and could be employed for evaluating associations for tumor-immune cell landscape and therapeutic effect [38, 39]. The scores of 22 human immune cell subpopulations from KIRC cases were calculated accurately through the CIBERSORT algorithm [40].

### 2.6. Definition and Assessment of a Novel Necroptosis-Associated lncRNA Expression Profile

All patients were segregated in a randomized manner within either a training group (*n* = 264) regarding necroptosis-associated lncRNA expression profile generation and a validating group (*n* = 262) regarding model validation, respectively. Univariate Cox regression assessment was executed within training group to bear necroptosis-associated prognostic lncRNAs. *p* < 0.05 was deemed to act as cutoff threshold for statistical significance. Through support by “glmnet” R package, LASSO regression analysis was employed for identifying ideal putative lncRNAs and construing a prognosis-associated expression profile. The following formula was employed:

(1) $$\begin{array}{c} \text{Risk Score}=\Sigma \left(\text{Expi}*\text{Coefi}\right), \end{array}$$

where Expi represents lncRNA expression level and Coefi reflects the expected regression coefficient for the individual lncRNA. All KRIC cases were placed into high- or low-risk groups, depending upon risk scoring. Survival assessment across both risk groups was performed for evaluating statistical significance in OS variations. Receiver operating characteristic (ROC) curves were designed through “survivalROC” in R, for evaluating prognosis properties by the survival model. In order to confirm prediction effectiveness by this model, the survival model was deployed onto the validation group and developed Kaplan–Meier survival/ROC curves accordingly.

### 2.7. Clinical Significance for Prognostic Risk Stratification

In order to evaluate prognosis value for necroptosis-associated lncRNA model in KIRC, the study focused on associations for risk-scoring and clinical/pathology features through univariate/multivariate Cox regression analyses. Subsequently, the hazard ratio (HR) with 95% confidence intervals and log-rank *p* value were determined through “glmnet” and “survival” packages in R [35].

A stratified assessment was employed for evaluating consistency in model prediction across multiple subgroups. Such parameters included age (≤ 65/> 65 years), gender (female/male), tumor stage (stage I/II/III/IV/unknow), grade (G1/G2/G3/G4/unknow), T stage (T1/T2/T3/T4), N stage (N0/N1/unknow), M stage (M0/M1/unknow), cluster (Cluster1/2), risk (high/low), and immune score (high/low). Furthermore, in order to examine expression profile influence upon clinical/pathology-based KIRC features, correlations between both such factors were evaluated through Chi-square test and presented through “pheatmap”/“ggpubr” R packages [41].

### 2.8. Gene Set Enrichment Analysis (GSEA)

(MSigDB, https://www.gsea-msigdb.org/gsea/msigdb) Genome-wide expression profiles for KRIC cases were assessed for GSEA in order to identify dysregulated genes across high-/low-risk group cases. The GSEA function in the Java software was executed, and the Hallmark gene set “c2.cp.kegg.v7.4.symbols.gmt” was used. Overall, 1000 random case permutations/enriched gene sets having *p* < 0.05 were encompassed within this evaluation, with FDR < 0.25 deemed to confer statistical significance. All remaining variables had default values.

### 2.9. Immunity Correlation Analysis

Meanwhile, the CIBERSORT [42], ESTIMATE [36], MCPcounter [43], single-sample gene set enrichment analysis (ssGSEA) [44], and TIMER algorithms [45] were comparatively analyzed for cell-based constituent components/immune repercussions across both risk groups, depending upon necroptosis-associated lncRNA model. Variations within immune-system repercussions by differing algorithms were revealed through a heat map. In addition, ssGSEA was employed for quantifying tumor-infiltrative immune cell subgroups across both groups together with evaluating immune effects. Possible immune-system checkpoints were also identified through past investigations.

### 2.10. Drug Sensitivity Analysis

In order to probe associations for necroptosis-associated lncRNA expression and anticancer drug sensitivity, the nine necroptosis-associated lncRNA expression and anticancer drug sensitivity datasets were collected through CellMiner (https://discover.nci.nih.gov/cellminer/home.do), followed by filtering anticancer drug sensitivity datasets (postclinical laboratory confirmation/FDA certifications). Subsequently, Pearson correlation analysis was employed for exploring such an association.

### 2.11. Quantitative Real-Time Polymerase Chain Reaction (qRT-PCR)

The above necroptosis-associated lncRNAs were verified using KIRC cell line (786-O, National Collection of Authenticated Cell Cultures, China) and human renal proximal convoluted tubule cell line (HK-2, National Collection of Authenticated Cell Cultures, China). Total cellular RNA was extracted from 786-O and HK-2 cells with TRIzol reagent (Beyotime, China) and reverse transcribed into cDNA. qPCR amplification was performed in CFX96 real-time PCR detection systems (Bio-Rad, USA) using the SYBR Green PCR kit (Servicebio, China). The primers are shown in Supplementary Table S1. GADPH was used as the internal control. The PCR parameters were set for an initial cycle of 1 minute at 95°C, followed by a total of 40 cycles at 95°C for 20 seconds, 55°C for 20 seconds, and 72°C for 30 seconds. The relative expression of each gene was calculated and compared using the 2*ΔΔ*Ct method. Experiments were repeated three times.

### 2.12. Statistical Analyses

All statistical evaluations employed R (version 4.0.4). For each analysis, *p* < 0.05 conferred statistical significance. Uni-/multivariate Cox proportional hazard regression analyses were used for determining necroptosis-associated lncRNA model use as a separate prognosis indicator. Chi-squared test was used to assess the medical features for differing study groups. Kaplan–Meier survival analyses were employed for the bilateral logarithmic rank test, assessing OS variations across KIRC cases. ROC was used for scoring prediction property sensitivity and specificity for this necroptosis-associated lncRNA prognostic model.

## 3. Results

### 3.1. KIRC Necroptosis-Associated lncRNA Recognition

**Figure 1:**
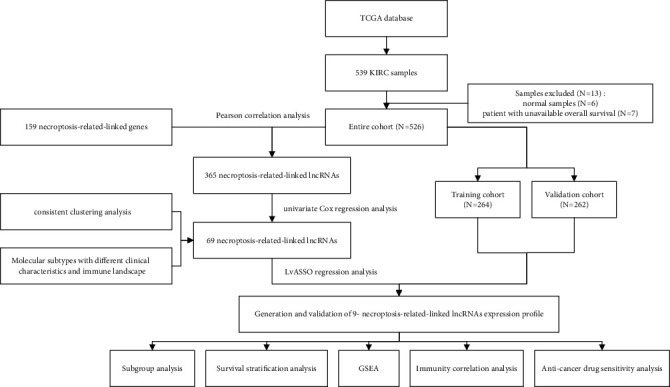
Schematic diagram for total analysis process.

**Figure 2:**
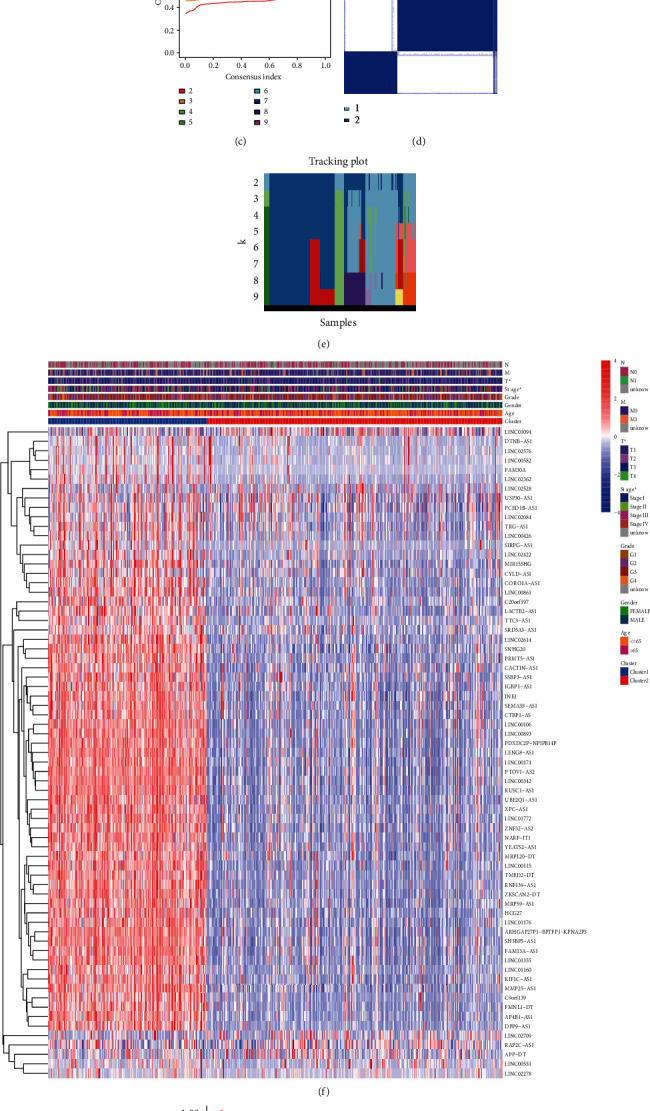
Consistent cluster analysis and differential clinical prognostic features in two KIRC clustering subgroups. (a) Forest plots of the relationship between 69 necroptosis-associated lncRNAs and the OS of KIRC. (b) The consensus clustering cumulative distribution function (CDF) for *k* = 2 to 9. (c) Relative change of the area under the CDF curve for *k* = 2 to 9. (d) Consensus clustering matrix for *k* = 2. (e) The distribution of the KIRC cases for *k* = 2 to 9. (f) Heat map of the two clustering subgroups with clinicopathologic features in KIRC. (g) Kaplan–Meier curves of OS for KIRC patients in two clustering subgroups.

Schematic diagram for the total analytical process is shown in Figure 1. Transcriptomic datasets for 539 KIRC cases/72 healthy volunteers through TCGA were investigated, leading to the identification of 4,668 lncRNAs. Overall, 159 necroptosis-associated genes were attained through KEGG (https://www.kegg.jp/entry/hsa04217; Supplementary Table S2). In addition, screening was performed on 1,210 necroptosis-associated lncRNAs that were intimately linked to necroptosis-associated genes through Pearson correlation analysis (|*R*| > 0.7 and *p* < 0.001). The 365 necroptosis-associated lncRNAs were differentially expressed between cancer and normal samples with statistical significance (Supplementary Table S3). Consequently, univariate Cox regression analytical dataset outcomes demonstrated 69 necroptosis-associated lncRNAs to be highly linked to KIRC case OS timeframes (Figure 2(a)) and were deemed to be utilized for further analyses in this study.

### 3.2. Consensus Clustering Discerned Differing Molecular-Based Subtypes

In order to recognize molecular-based subtypes, this study conducted consensus clustering to classify 69 necroptosis-associated prognostic lncRNAs by using *k*-means algorithm. The consensus clustering results were compared using differing *K* values. Finally, *k* = 2 was selected as the ideal cluster quantity for additional investigations stemming from minimized interferences across both subgroups (Figures 2(b) and 2(c)). A total of 526 KIRC cases were stratified within both subgroups called cluster 1 (*n* = 184) and cluster 2 (*n* = 342) (Figures 2(b)–2(e)).

### 3.3. Molecular Subtypes with Different Clinical Characteristics and Immune Landscape

Necroptosis-associated lncRNA within cluster 2 was downregulated, including PCED1B-AS1, LINC00426, LACTB2-AS1, LINC02422, and LINC00861 (Figure 2(f)). Additionally, clinicopathological features across subgroups were recognized through consensus clustering. Dataset outcomes showed cluster 1 to be predominantly linked to tumor T stage (*p* < 0.05; Figure 2(f)) and advanced tumor stage (*p* < 0.05; Figure 2(f)). Besides those results, OS variations were analyzed across subgroups. Patients with cluster 2 have better survival than those with cluster 1 (*p* < 0.001; Figure 2(g)). Collectively, clustering subgroups were highly associated with KIRC heterogeneity.

**Figure 3:**
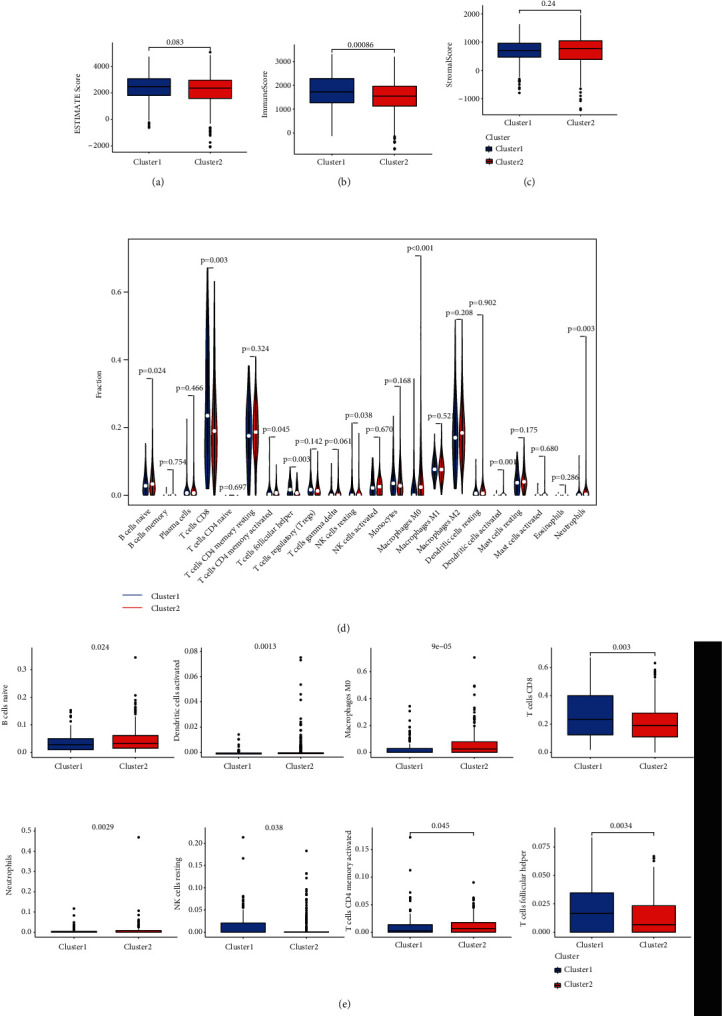
The TME scores and scores of 22 human immune cell subpopulations in two KIRC clustering subgroups. (a–c) The immune score, interstitial score, and tumor purity of two clustering subgroups. (d) The difference of 22 human immune cell subpopulations between clusters I and II. (e) The infiltrative levels of naïve-B cells, activated dendritic cells, M0-level-macrophages, CD8-T cells, neutrophils, resting-NK cells, activated CD4 memory-T cells, and follicular-helper-T cells between two KIRC clusters were significantly different.

In order to assess necroptosis-associated lncRNA functions over KIRC TME, the study analyzed stromal, immune, and estimate scorings for both subgroups and determined the degree for 22 immune cell types. Dataset outcomes demonstrated revealed stromal and immune scorings to be highly raised within cluster 1, while stromal scoring was raised within cluster 2 (Figures 3(a)–3(c)). As a result, cluster 1 had increased infiltrative levels of CD8-T cells, follicular-helper-T cells, and resting-NK cells, with increased levels of naïve-B cells, activated CD4 memory-T cells, M0-level-macrophages, activated-dendritic cells, and neutrophils were identified within cluster 2 (*p* < 0.05; Figures 3(d) and 3(e)).

### 3.4. Generation and Validation for Necroptosis-Associated lncRNA Prognostic Model

According to the results of Chi-square test, there was no significant variation across both training and verification groups throughout all comparisons (*p* > 0.05; Supplementary Table S4).

**Figure 4:**
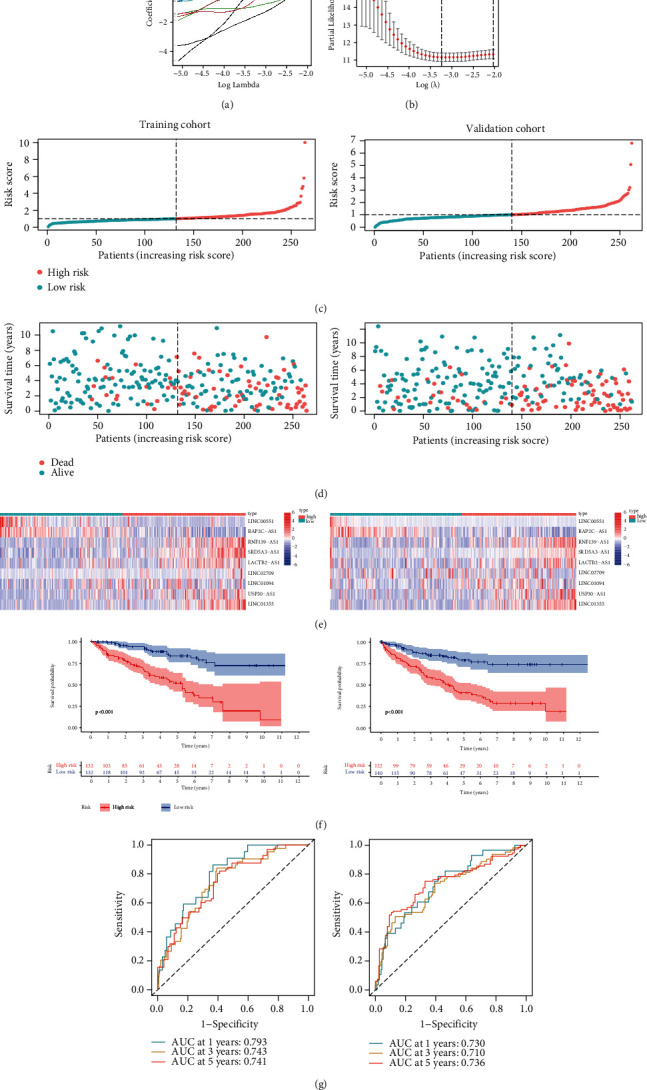
The prognostic value of the necroptosis-associated lncRNA model in the training cohort and validation cohort. (a) LASSO coefficient profiles of nine necroptosis-associated lncRNAs. (b) Nine necroptosis-associated lncRNAs were included when the cross-validation error in the LASSO model was minimal. (c) The risk score distribution of the low-/high-risk KIRC groups in the training and validation cohorts. (d) Survival status of KIRC patients with low-/high-risk scores in the training and validation cohorts. (e) Heat map of a nine necroptosis-associated lncRNA model in the training and validation cohorts. (f) Kaplan–Meier survival curves for KIRC patients in the low-/high-risk KIRC groups in the training and validation cohorts. (g) ROC curves and AUCs of the nine necroptosis-associated lncRNA model in the training and validation cohorts.

In order to minimize overfitting across prognosis-based biomarkers, LASSO Cox analysis was employed for further analysis of 69 necroptosis-associated prognostic lncRNAs. Finally, 9 lncRNAs (RNF139-AS1, SRD5A3-AS1, LINC00551, RAP2C-AS1, LACTB2-AS1, LINC02709, LINC01094, USP30-AS1, and LINC01355) were identified from the training group (Figures 4(a) and 4(b)). Subsequently, a nine-necroptosis-associated prognostic lncRNA model was determined through modified regression coefficients for individual lncRNAs, with risk scorings determined depending upon summated expression levels for RNF139 − AS1∗0.500 + SRD5A3 − AS1∗1.193 − LINC00551∗1.556 − RAP2C − AS1∗0.900 − LACTB2 − AS1∗0.411 + LINC02709∗0.098 + LINC01094∗0.032 + USP30 − AS1∗0.028 + LINC01355∗0.158. After calculating individual case risk scoring using the prognostic model, KIRC cases were segregated within the low-/high-risk groups depending upon median risk scoring ranks, based upon the expression profile model (Figure 4(c)). Scatter plots reflected OS for KIRC cases depending upon risk scoring and showed that the high-risk scoring group was intimately linked to mortality rate (Figure 4(e)). The expression profiles for the nine lncRNAs suggested that tumors having elevated risk scores had overall upregulation for RNF139-AS1, SRD5A3-AS1, LINC02709, LINC01094, USP30-AS1, LINC01355, and downregulation for RAP2C-AS1, LINC00551, LACTB2-AS1 (Figure 4(e)). Kaplan–Meier plots demonstrated that the high-risk group cases had significantly poorer OS than low-risk cases within the training group (*p* < 0.05; Figure 4(f)). ROC curve analyses suggested that the survival model applied to the training group had sufficient prediction properties. The AUC at 1 year for ROC curves was 0.793. The AUC at 3 years for ROC curves was 0.743. The AUC at 5 years for ROC curves was 0.741 (Figure 4(g)). In order to validate that the necroptosis-associated model possessed reliable prognosis prediction worth, an identical evaluation was conducted across the validation group, whereby the dataset outcomes were in line with those stemming from the training group (Figures 4(c)–4(g)). In a similar manner, variations in Kaplan–Meier survival curve across both risk groups carried statistical significance within the validation group (Figure 4(f)). AUC for ROC curve evaluations was 0.730 at one year. AUC for ROC curve evaluations was 0.710 at three years. AUC for ROC curve evaluations was 0.736 at five years (Figure 4(g)).

### 3.5. Subgroup Analyses with Different Clinicopathological Features

**Figure 5:**
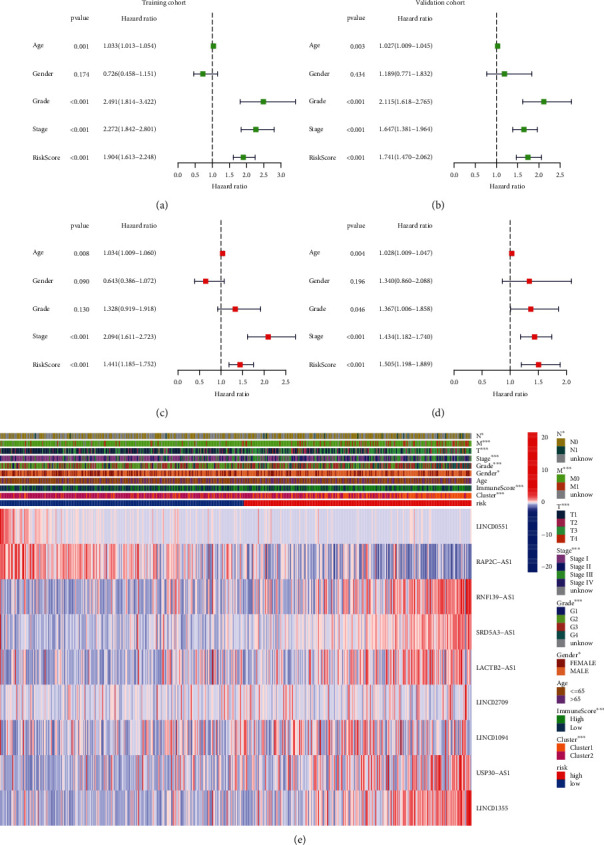
Relationship between the high-/low-risk score and clinicopathological features. (a, b) Forest plots of factors associated with OS by univariate Cox regression analysis in the training and validation cohort. (c, d) Forest plots of factors associated with OS by multivariate Cox regression analysis in the training and validation cohorts. (e) Heat map showed the significant difference of ages, TNM stage, tumor stage, tumor grade, gender, and immune scores in the high-/low-risk KIRC groups.

Consequently, uni-/multivariate Cox regression analyses were conducted for discerning if such a lncRNA model could serve as a separate prognosis indicator. Within the training group, the risk scoring and tumor stage were highly linked to OS across both Cox regression analyses types (*p* < 0.001; Figures 5(a) and 5(c)). Notably, risk scoring/tumor stage was also intimately linked to OS within the validation group through identical analyses, suggesting that risk scoring acted as a separate robust OS-prognostic parameter for KIRC (Figures 5(b) and 5(d)).

The expression of nine necroptosis-associated prognostic lncRNAs together with the spread of clinical/pathological features, TME immune scorings, together with case clustering into the high-/low-risk groups was visualized through a heat map (Figure 5(e)). Distinct variations across both groups depending upon differing clusters (*p* < 0.001) were identified. Major variations in risk scoring were identified across differing ages (*p* < 0.05), TNM stage (*p* < 0.05), tumor stage (*p* < 0.05), tumor grade (*p* < 0.05), gender (*p* < 0.05), and immune scores (*p* < 0.001).

### 3.6. Survival Stratification Analysis

**Figure 6:**
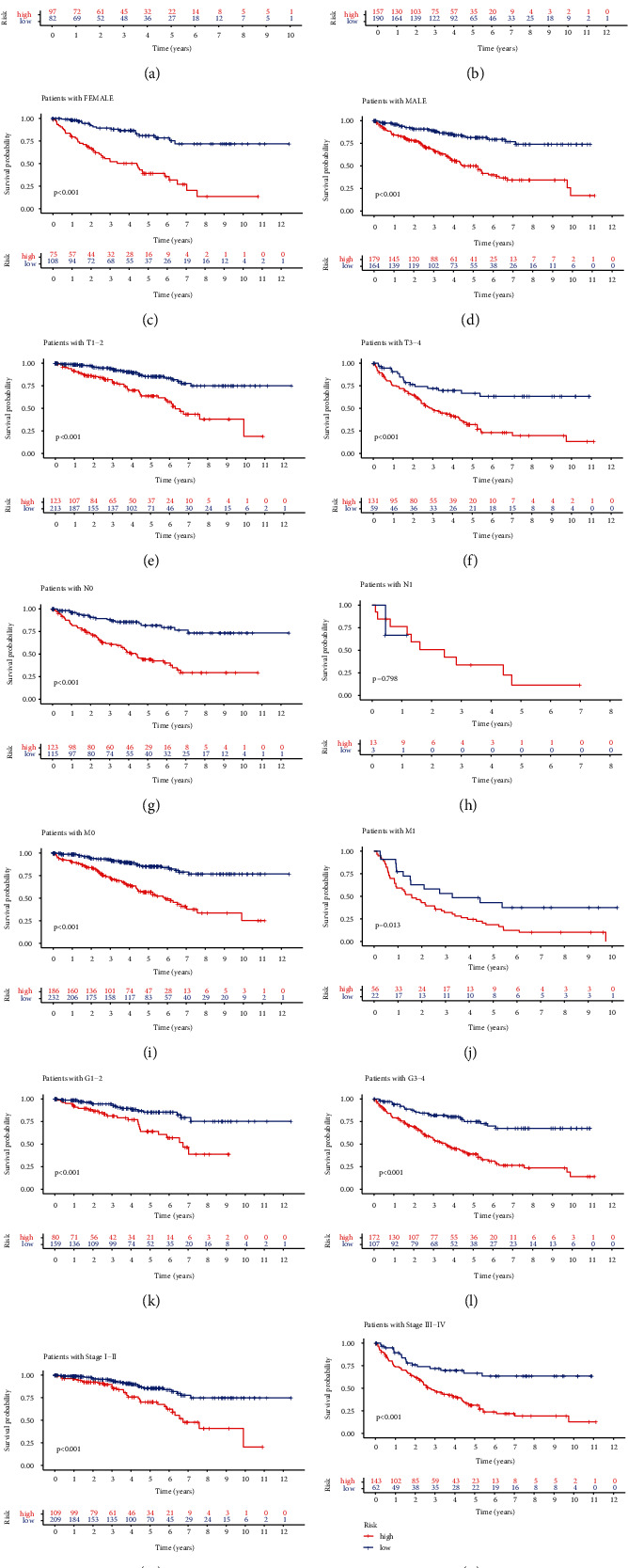
Survival stratification analysis of the necroptosis-associated lncRNA model in KIRC: (a) age > 65; (b) age ≤ 65; (c) female; (d) male; (e) T1-T2; (f) T3-T4; (g) N0; (h) N1; (i) M0; (j) M1; (k) G1–G2; (l) G3-G4; (m) stages I–II; (n) stages III–IV.

In order to evaluate whether the model has predictive ability in KRIC case subgroups having differing medical features, subgroups were stratified through age (age > 65/age ≤ 65), gender (female/male), stage T (T1-T2/T3-T4), stage N (N0/N1), stage M (M0/M1), grade (G1-G2/G3-4), and clinical stage (stages I-II/III-IV). Illustrated in Figure 6, dataset outcomes demonstrated that low-risk cases depending upon age (*p* < 0.001 in age ≤ 65 and *p* < 0.001 in age > 65), sex (*p* < 0.001 in female and *p* < 0.001 in male), stage T (*p* < 0.001 in T1-T2 and *p* < 0.001 in T3-T4), stage N0 (*p* < 0.001), stage M (*p* < 0.001 in M0 and *p* = 0.013 in M1), and clinical stage (*p* < 0.001 in stages I–II and *p* < 0.001 in stages III–IV) had the best prognostic odds.

### 3.7. Gene Set Enrichment Analysis

**Figure 7:**
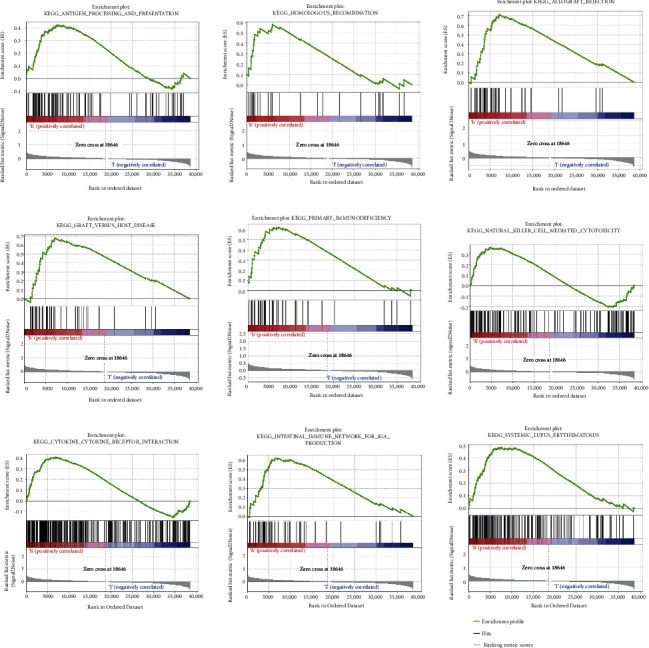
Gene set enrichment analysis (GSEA) of the high-/low-risk KIRC groups based on the necroptosis-associated lncRNA prognostic model.

This was employed for rooting out the major physiological roles adopted by the nine necroptosis-associated lncRNA model. The results revealed that the necroptosis-associated lncRNA prognostic model modulated immune-associated disease and processes such as antigen processing and presentation, homologous recombination, allograft rejection, graft versus host disease, primary immunodeficiency, NK cell-mediated cytotoxicity, cytokine receptor interaction, intestine-based immune-networking for IgA generation, and systemic lupus erythematosus (Figure 7).

### 3.8. Immunity Correlation Analysis of Necroptosis-Associated lncRNA Model

**Figure 8:**
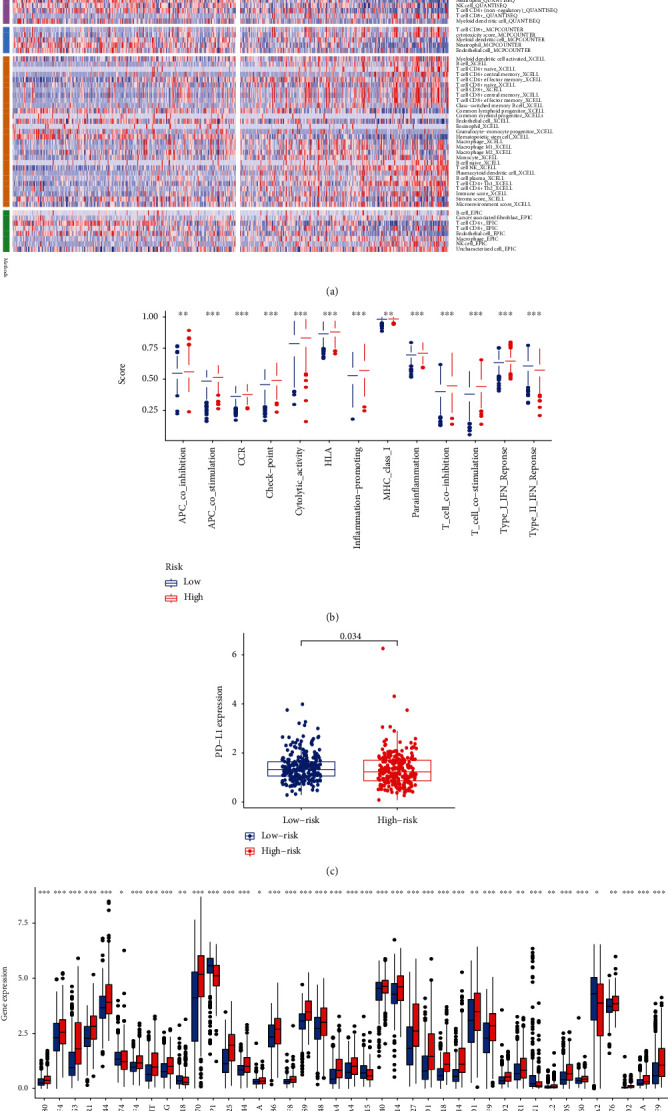
Immunity correlation analysis of the necroptosis-associated lncRNA model in KIRC. (a) Heat map of immune cell infiltration landscape in the high-/low-risk KIRC groups. (b) ssGSEA for the relationship between immune functions and immune cell subpopulations in the low-/high-risk KIRC groups. (c) The expression levels of PD-L1 between high-/low-risk KIRC groups. (d) The expression levels of immune checkpoints between high-/low-risk KIRC groups.

Depending upon CIBERSORT, CIBERSORT−ABS, QUANTISEQ, MCPCOUNTER, XCELL, EPIC, and TIMER algorithms, we scrutinized the immune cell and pathway profiles among both groups (Figure 8(a)). The ssGSEA highlighted distinct variations regarding T cell function (Figure 8(b)), with the high-risk group having higher scores for coinhibition, costimulation, CCR, checkpoint, cytolytic activity, HLA, inflammation-enhancing, MHC-class-I, para-inflammation, and type-I-IFN-response. Consequently, we further investigated the dysregulated expression of immune checkpoints across both risk groups, especially for PD-L1 expression. Illustrated within Figure 8(c), the KRIC risk groups had distinct variations in PD-1 expression (*p* = 0.034). Moreover, both groups showed distinct dysregulations within immune checkpoint expression and multiple immune checkpoints being upregulated (CD274, LAG3, CTLA4, BTLA, and PDCD1) within the high-risk group (Figure 8(d)).

### 3.9. Anticancer Drug Sensitivity Analysis of Necroptosis-Associated lncRNAs

**Figure 9:**
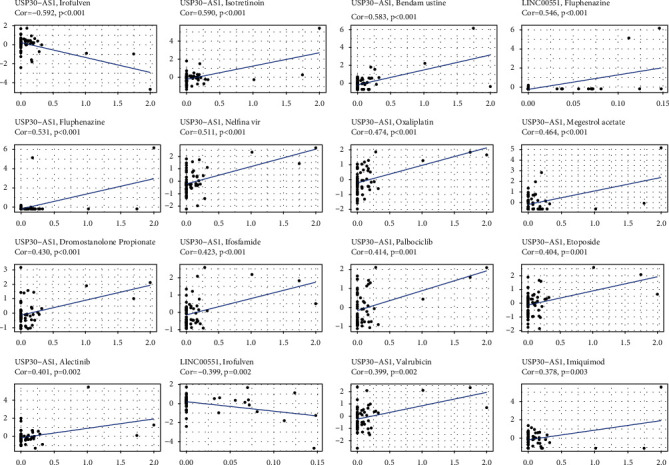
Anticancer drug sensitivity analysis of necroptosis-associated lncRNAs.

Since necroptosis-associated lncRNAs are often associated with stem cell-like features, we further studied the expression of necroptosis-associated lncRNAs in cancer cell lines and then comprehensively analyzed the correlation between their expression levels in cancer cell lines with drug sensitivity of >200 chemotherapeutic drugs. We observed that the expression levels of necroptosis-associated lncRNAs showed great heterogeneity in cancer cell lines and in cancer patients. Interestingly, we found that increased expression of necroptosis-associated lncRNAs was related to increased drug resistance to a variety of chemotherapy drugs in cancer cell lines. As shown in Figure 9, the expression of USP30-AS1 was positively correlated with the sensitivity of isotretinoin, bendamustine, fluphenazine, nelfinavir, oxaliplatin, megestrol acetate, dromostanolone pro, ifosfamide, palbociclib, etoposide, alectinib, valrubicin, and imiquimod. The expression of USP30-AS1 had a remarkable negative relationship with the sensitivity of irofulven. Upregulated LINC00551 led to enhanced drug sensitivity for fluphenazine, though it was also associated with reduced drug sensitivity for irofulven.

### 3.10. The Results of qRT-PCR

**Figure 10:**
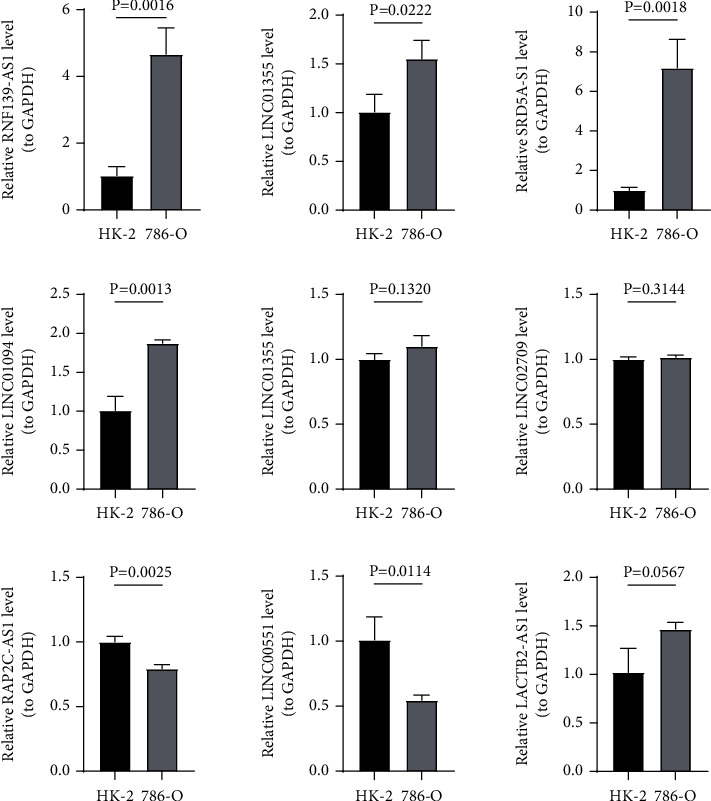
The qRT-PCR results of necroptosis-associated lncRNA relative expressing levels in two lineage cells (HK-2 and 786-O).

In addition, HK-2 and 786-O cell lines were used to verify the above necroptosis-associated lncRNAs. The results showed that the expressions of RNF139-AS1, SRD5A3-AS1, LINC01094, USP30-AS1, LACTB2-AS1, and LINC01355 were elevated in renal carcinoma cells, and the expressions of RAP2C-AS1 and LINC00551 were reduced in renal carcinoma cells compared with normal renal proximal convoluted tubule cells (Figure 10).

## 4. Discussion

KIRC is a major malignant tumor of the urinary system, and approximately 33% of patients have metastasis when diagnosed. Although treatment methods have advanced recently, relapse/mortality rates are still very high, especially for advanced and metastatic patients, where the prognosis is poor [46]. Recent studies suggest that tumor cells resistant to apoptosis may be sensitive to the necroptosis pathway [24, 25], suggesting that necroptosis may be a potential therapeutic target for KIRC. Consequently, it is vital to develop biomarkers for early diagnosis, treatment, and prognosis monitoring of KIRC patients.

This investigation identified 9 novel necroptosis-associated prognostic lncRNA expression profile by Pearson's correlation analysis between necroptosis-associated genes and lncRNAs in KIRC cases obtained from TCGA datasets. Subsequently, we divided 526 KIRC samples into two cluster subgroups, and both demonstrated distinctly differing survival, clinical features, immune score, and immune cell infiltrative properties. It is also in line with previous similar studies [47, 48]. Subsequently, LASSO Cox regression analysis was conducted, with a nine necroptosis-associated lncRNA prognostic expression profile being generated. Among the nine necroptosis-associated lncRNAs, six were unfavorable diagnostic factors for KIRC (RNF139-AS1, SRD5A3-AS1, LINC02709, LINC01094, USP30-AS1, and LINC01355), and three were favorable diagnostic factors for KIRC (LINC00551, RAP2C-AS1, and LACTB2-AS1). The expression profile was employed to categorize KIRC patients into the high-risk and low-risk groups, depending upon median-risk score. A high-risk score was linked to poor OS/late-stage clinicopathological features. The predictive power of this expression profile was validated through the ROC curve and the validation group. Cases across both risk groups showed distinctly differing molecular-interplay profiles, PD-L1 expression, and immune score. In addition, a stratified analysis showed that the expression profile retains its prediction power across differing subgroups. Multivariate analysis suggested that the expression profile was also a separate indicator in comparison to alternative clinicopathological features. In conclusion, the assembly of markers—including nine necroptosis-associated lncRNAs—proved to be prognostic biomarkers for KIRC. Compared with other prognostic models, our prognostic model focuses on necroptosis-associated lncRNAs and could be employed for prognostic stratification of KIRC cases and contributing novel drug targeting therapeutic options and provide new theoretical foundations and treatment options for KIRC. In addition, these necroptosis-associated lncRNAs may play an important role in the prognosis of KIRC by targeting microRNAs and mRNAs, which may also better reveal their molecular mechanisms.

Presently, many studies have demonstrated the crucial role of lncRNAs in the necroptosis of malignant tumor cells. Min et al. [49] found that lncRNA CRLA was significantly associated with EMT and chemotherapy resistance of lung adenocarcinoma cells. By binding to the intermediate domain of RIPK1, it weakens the interaction of RIPK1-RIPK3, thus significantly upregulating and inhibiting RIPK1-induced necroptosis. However, the research reports of necroptosis-associated lncRNAs in relation to cancer—and especially KIRC—are extremely inadequate. A large number of preceding studies have shown that lncRNAs play a key role in the occurrence and development of cancer by regulating corresponding miRNAs and target genes. For this reason, it is necessary to explore the role of gene-related lncRNA in tumors. We conducted a correlation analysis between necroptosis-associated genes and lncRNAs to determine necroptosis-associated lncRNAs. The recommended prognostic expression profile contained nine necroptosis-associated lncRNAs in this study. Among these nine lncRNAs, the specific mechanisms of SRD5A3-AS1, RNF139-AS1, LACTB2-AS1, RAP2C-AS1, and LINC02709 have not yet been reported. The results of many previous studies are identical to our analysis, showing that LINC00551 acts as a tumor suppressor. Wang et al. [50] found that LINC00551 was differentially downregulated in lung adenocarcinoma, and its expression level correlated with clinical prognosis. LINC00551 inhibits glycolysis and tumor progression by regulating the expression of PKM2, which is mediated by c-Myc in pulmonary adenocarcinoma. LINC00551 binds to HSP27 to reduce its level of phosphorylation, thereby downregulating the proliferation and invasion of esophageal squamous cell carcinoma cells [51]. LINC00511 is upregulated in non-small-cell lung cancer tissues and cell lines. LINC00511 downregulates LATS2 and KLF2 by combining EZH2 and LSD1 to promote the proliferation, migration, and invasion of non-small-cell lung cancer [52]. One past study has shown that LINC01094 activates radio-resistance of clear cell RCC through miR-577/CHEK2/FOXM1 axis [53]. Moreover, robust evidence has shown that there is an intimate relationship between LINC01094 and prognosis in KIRC [54, 55]. LINC01355 has differing effects on different tumors. Conversely, LINC01355 inhibits the growth of breast cancer by inhibiting FOXO3-mediated CCND1 transcription [56]. Conversely, it activates the Notch signaling pathway to promote the malignant phenotype of oral squamous cells and the invasion of cytotoxic T cells [57]. Currently, many studies have found a close relationship between USP30-AS1 and autophagy in cancer [58–61], though there is no study on the relationship between USP30-AS1 and necroptosis. However, further studies are needed to verify the specific mechanism of these lncRNAs in tumors.

Nevertheless, our present study contains a few limitations. Firstly, it is not comprehensive enough to only use bioinformatics methods and public databases for analysis. More basic studies are still needed to explore the mechanism of necroptosis-associated lncRNAs in the progress of KIRC. Secondly, our study clarified the correlation between necroptosis-associated lncRNAs and KIRC tumor immune status, laying a theoretical foundation for the enhancement of antitumor immunity and novel therapeutic targets of KIRC, though the specific mechanism of immunity remains to be further revealed. Thirdly, although our expression profile profiles were validated in the TCGA validation group, we should be cautious in assessing the prognostic value of necroptosis-associated lncRNA expression profile. Therefore, further validation with larger clinical samples is required to verify these results.

In conclusion, we assessed survival, clinical characters, and immune cell infiltration levels in two clustered subgroups and constructed a nine necroptosis-associated lncRNA prognostic expression profile in KIRC, which had significant value in predicting the OS of patients with KIRC, clinicopathological characteristics, TME, immune score, and anticancer drug sensitivity. Additionally, we found a close correlation between necroptosis-associated lncRNAs and KIRC drug sensitivity, which paves the way to augment antitumor immunity and novel therapeutic systems for KIRC. This work also provides important evidence for the development of predictive biomarkers and immunotherapy for KIRC.

## Data Availability

This study used and analyzed currently publicly available datasets. These data can be found in the article/supplementary material.

## Supplementary Materials

Supplementary 1 Supplementary Table S1: the primers used in this study for RT-PCR.

Supplementary 2 Supplementary Table S2: summary of 159 necroptosis-associated genes.

Supplementary 3 SupplementaryTable S3: summary of 365 necroptosis associated lncRNA.

Supplementary 4 Supplementary Table S4: clinicopathological features of KIRC patients between the training and verification groups.
