# Supplementary material for: Clinicopathological and Prognostic Value of Necroptosis-Associated lncRNA Model in Patients with Kidney Renal Clear Cell Carcinoma
Source: Dis Markers. 2022 May 23;2022:5204831. doi: 10.1155/2022/5204831 (PMC9157284; doi:10.1155/2022/5204831)
Supplement: Supplementary 1 — Supplementary Table S1: the primers used in this study for RT-PCR. [file 5204831.f1.docx]

Supplementary Table S1: The primers used in this study for RT-PCR.

RNF139-AS1 Forward:5′-CTTGCGATGGACAGAAGTGAG-3′ Reverse:5′-ATCTCCTCTACCCAACTTGTGT-3′

SRD5A3-AS1 Forward:5′-CAACACAGCAAGACCTTGATTC-3′ Reverse:5′-AGACCTGGATGGCATTGAGT-3′

LINC02709 Forward:5′-GAGTGAGATTATGGCGAAGCAT-3′ Reverse:5′-CATTCAAGGCTACTACTGGAGAG-3′

LINC01094 Forward:5′-TCCCTTCCACAGAGAAGGCT-3′ Reverse:5′-AGGTTGACACATCTCGCCTG-3′

USP30-AS1 Forward:5′-GTCTCCCCAGGTCTGTGCTTAA-3′

Reverse:5′-GTATTTTTTCCTTATGCTGCCAAA-3′ LINC01355 Forward:5′-CTGCTCTAGCCCCTAAAGATAG-3′

Reverse:5′-GGATTCCAAATGACACATTCCT-3′

RAP2C-AS1 Forward:5′-CAAGGGTGCCTTTTTGGAGC-3′ Reverse:5′-AAGAGCTTGATGACTCCGGC-3′

LINC00551 Forward:5′‐CAGCCTTCAGTTGGAGGAAC‐3′ Reverse:5′‐TGGCCAATGACGAATACTGA‐3′

LACTB2-AS1 Forward:5′‐CCACACTCTCATTCCACCAT-3′ Reverse:5′‐GGCAAGGGCTGTTATATCCA‐3′

GAPDH Forward:5′‐CCCATCACCATCTTCCAGG-3′

Reverse: 5′‐CATCACGCCACAGTTTCCC‐3′
