# Supplementary material for: Clinicopathological and Prognostic Value of Necroptosis-Associated lncRNA Model in Patients with Kidney Renal Clear Cell Carcinoma
Source: Dis Markers. 2022 May 23;2022:5204831. doi: 10.1155/2022/5204831 (PMC9157284; doi:10.1155/2022/5204831)
Supplement: Supplementary 2 — Supplementary Table S2: summary of 159 necroptosis-associated genes. [file 5204831.f2.docx]

Supplementary Table S2: Summary of 159 necroptosis-linked genes.

| Necroptosis-linked genes |
| --- |
| AIFM1 |
| ALOX15 |
| BAX |
| BCL2 |
| BID |
| BIRC2 |
| BIRC3 |
| CAMK2A |
| CAMK2B |
| CAMK2D |
| CAMK2G |
| CAPN1 |
| CAPN2 |
| CASP1 |
| CASP8 |
| CFLAR |
| CHMP1A |
| CHMP1B |
| CHMP2A |
| CHMP2B |
| CHMP3 |
| CHMP4A |
| CHMP4B |
| CHMP4C |
| CHMP5 |
| CHMP6 |
| CHMP7 |
| CYBB |
| CYLD |
| DNM1L |
| EIF2AK2 |
| FADD |
| FAF1 |
| FAS |
| FASLG |
| FTH1 |
| FTL |
| GLUD1 |
| GLUD2 |
| GLUL |
| H2AB1 |
| H2AB2 |
| H2AB3 |
| H2AC1 |
| H2AC11 |
| H2AC12 |
| H2AC13 |
| H2AC14 |
| H2AC15 |
| H2AC16 |
| H2AC17 |
| H2AC18 |
| H2AC19 |
| H2AC20 |
| H2AC21 |
| H2AC4 |
| H2AC6 |
| H2AC7 |
| H2AC8 |
| H2AJ |
| H2AW |
| H2AX |
| H2AZ1 |
| H2AZ2 |
| HMGB1 |
| HSP90AA1 |
| HSP90AB1 |
| IFNA1 |
| IFNA10 |
| IFNA13 |
| IFNA14 |
| IFNA16 |
| IFNA17 |
| IFNA2 |
| IFNA21 |
| IFNA4 |
| IFNA5 |
| IFNA6 |
| IFNA7 |
| IFNA8 |
| IFNAR1 |
| IFNAR2 |
| IFNB1 |
| IFNG |
| IFNGR1 |
| IFNGR2 |
| IL1A |
| IL1B |
| IL33 |
| IRF9 |
| JAK1 |
| JAK2 |
| JAK3 |
| JMJD7-PLA2G4B |
| MACROH2A1 |
| MACROH2A2 |
| MAPK10 |
| MAPK8 |
| MAPK9 |
| MLKL |
| NLRP3 |
| PARP1 |
| PGAM5 |
| PLA2G4A |
| PLA2G4B |
| PLA2G4C |
| PLA2G4D |
| PLA2G4E |
| PLA2G4F |
| PPIA |
| PPID |
| PYCARD |
| PYGB |
| PYGL |
| PYGM |
| RBCK1 |
| RIPK1 |
| RIPK3 |
| RNF103-CHMP3 |
| RNF31 |
| SHARPIN |
| SLC25A31 |
| SLC25A4 |
| SLC25A5 |
| SLC25A6 |
| SMPD1 |
| SPATA2 |
| SPATA2L |
| SQSTM1 |
| STAT1 |
| STAT2 |
| STAT3 |
| STAT4 |
| STAT5A |
| STAT5B |
| STAT6 |
| TICAM1 |
| TICAM2 |
| TLR3 |
| TLR4 |
| TNF |
| TNFAIP3 |
| TNFRSF10A |
| TNFRSF10B |
| TNFRSF1A |
| TNFSF10 |
| TRADD |
| TRAF2 |
| TRAF5 |
| TRPM7 |
| TYK2 |
| USP21 |
| VDAC1 |
| VDAC2 |
| VDAC3 |
| VPS4A |
| VPS4B |
| XIAP |
| ZBP1 |
