# Supplementary material for: Clinicopathological and Prognostic Value of Necroptosis-Associated lncRNA Model in Patients with Kidney Renal Clear Cell Carcinoma
Source: Dis Markers. 2022 May 23;2022:5204831. doi: 10.1155/2022/5204831 (PMC9157284; doi:10.1155/2022/5204831)
Supplement: Supplementary 3 — SupplementaryTable S3: summary of 365 necroptosis associated lncRNA. [file 5204831.f3.docx]

Supplementary Table S3: Summary of 365 necroptosis-linked lncRNAs.

| lncRNAs | pValue |
| --- | --- |
| CELF2-AS1 | 7.91E-17 |
| BMPR1B-DT | 4.55E-52 |
| LINC00551 | 1.62E-36 |
| LINC02340 | 5.71E-05 |
| MDS2 | 1.42E-28 |
| NAV2-AS3 | 2.48E-38 |
| LCA10 | 3.80E-19 |
| PRICKLE2-AS3 | 2.68E-09 |
| LINC00967 | 1.23E-13 |
| LINC01732 | 8.13E-22 |
| TBL1XR1-AS1 | 1.75E-11 |
| IGBP1-AS1 | 7.08E-17 |
| SAMD12-AS1 | 4.07E-23 |
| RAP2C-AS1 | 1.23E-33 |
| RNF139-AS1 | 7.27E-06 |
| RNASEH2B-AS1 | 6.75E-05 |
| LINC00342 | 6.71E-27 |
| LINC01182 | 4.64E-29 |
| KANSL1L-AS1 | 6.21E-14 |
| LINC02345 | 8.19E-18 |
| LINC01684 | 6.45E-27 |
| LINC01230 | 2.60E-39 |
| LINC02588 | 0.038513155 |
| LINC00106 | 1.03E-11 |
| SOX2-OT | 2.15E-31 |
| CERS6-AS1 | 0.00021047 |
| PRKCA-AS1 | 0.000448646 |
| LINC00486 | 0.026296549 |
| BACH1-IT2 | 8.81E-10 |
| ST3GAL3-AS1 | 3.60E-09 |
| LINC02579 | 4.12E-09 |
| C7orf65 | 1.64E-07 |
| LINC01290 | 3.80E-05 |
| PACRG-AS1 | 2.78E-27 |
| LINC02097 | 8.40E-20 |
| LINC01931 | 0.015208874 |
| EML2-AS1 | 3.63E-10 |
| EAF1-AS1 | 8.16E-09 |
| INSYN1-AS1 | 4.63E-38 |
| ARHGEF26-AS1 | 2.71E-32 |
| ZKSCAN2-DT | 1.06E-16 |
| IFT74-AS1 | 8.16E-23 |
| LINC01976 | 6.44E-51 |
| LINC02084 | 9.70E-27 |
| LINC01979 | 1.49E-06 |
| LINC01879 | 1.45E-24 |
| LINC01160 | 1.33E-11 |
| MCF2L-AS1 | 1.59E-36 |
| ZNF628-DT | 1.76E-05 |
| LINC01907 | 7.64E-13 |
| NARF-IT1 | 1.37E-17 |
| DAPK1-IT1 | 5.81E-08 |
| MPPED2-AS1 | 4.36E-41 |
| LINC01722 | 2.84E-07 |
| LINC02533 | 4.59E-08 |
| KCNIP1-OT1 | 0.013513299 |
| LINC02576 | 9.85E-08 |
| CORO1A-AS1 | 1.21E-29 |
| SEMA3F-AS1 | 2.61E-11 |
| LINC01775 | 1.57E-06 |
| LINC01968 | 1.86E-10 |
| LINC02078 | 3.56E-13 |
| LINC01664 | 3.17E-16 |
| LINC00582 | 4.11E-05 |
| LINC01891 | 4.14E-15 |
| LINC01447 | 5.44E-30 |
| FAM30A | 1.35E-10 |
| AP4B1-AS1 | 3.36E-15 |
| TRG-AS1 | 1.82E-35 |
| LINC02568 | 6.99E-38 |
| MYCNOS | 1.94E-20 |
| SRD5A3-AS1 | 7.86E-30 |
| LINC02574 | 1.87E-07 |
| LINC00265 | 1.47E-07 |
| LINC00410 | 1.29E-17 |
| RUFY1-AS1 | 1.94E-22 |
| PLCH1-AS1 | 0.024208042 |
| LINC02133 | 0.000115638 |
| FAM230I | 7.27E-20 |
| LINC01087 | 2.81E-08 |
| SPATA3-AS1 | 0.001126381 |
| LACTB2-AS1 | 6.71E-29 |
| LINC01356 | 1.09E-25 |
| SKAP1-AS1 | 3.54E-05 |
| LINC00461 | 7.38E-35 |
| LINC01227 | 1.38E-08 |
| LINC02585 | 8.69E-11 |
| PRKX-AS1 | 1.89E-05 |
| ZFHX2-AS1 | 4.35E-16 |
| LINC02354 | 1.65E-19 |
| HCG27 | 3.72E-32 |
| LINC01833 | 7.57E-25 |
| LINC01706 | 1.39E-23 |
| FAM215B | 0.002989335 |
| FGF12-AS2 | 8.70E-18 |
| MTUS2-AS1 | 8.49E-10 |
| SRGAP3-AS4 | 2.88E-45 |
| IGBP1-AS2 | 3.97E-09 |
| CFAP20DC-AS1 | 3.08E-15 |
| LINC00311 | 0.000818513 |
| RABGAP1L-DT | 1.98E-22 |
| INE1 | 8.66E-11 |
| LINC01517 | 2.65E-08 |
| LINC01163 | 7.64E-23 |
| LINC02544 | 7.73E-13 |
| APP-DT | 6.12E-23 |
| DPP9-AS1 | 3.88E-32 |
| HPN-AS1 | 4.27E-08 |
| SCAT1 | 2.03E-26 |
| LINC02343 | 2.74E-54 |
| LINC01751 | 5.76E-32 |
| VIPR1-AS1 | 3.23E-10 |
| LINC02484 | 3.17E-16 |
| LINC01991 | 6.39E-23 |
| RMDN2-AS1 | 0.032658728 |
| CAVIN2-AS1 | 0.021900761 |
| GTF3C2-AS1 | 1.86E-14 |
| UBE2Q1-AS1 | 6.45E-19 |
| LINC02836 | 1.95E-09 |
| RBM38-AS1 | 1.17E-16 |
| LINC01786 | 1.06E-18 |
| LINC01012 | 2.01E-23 |
| C9orf139 | 1.24E-26 |
| LINC02080 | 1.18E-10 |
| LINC02356 | 7.21E-06 |
| FRY-AS1 | 9.93E-27 |
| TTC3-AS1 | 1.57E-06 |
| XPC-AS1 | 1.27E-12 |
| LINC00454 | 2.81E-05 |
| LINC01975 | 1.18E-37 |
| LENG8-AS1 | 3.37E-22 |
| LINC01280 | 5.07E-12 |
| LINC01623 | 8.28E-06 |
| EXTL3-AS1 | 0.003095565 |
| LINC02436 | 3.45E-11 |
| GAS6-DT | 8.73E-25 |
| LINC02177 | 8.43E-09 |
| CDC42-IT1 | 0.000395643 |
| NFIA-AS1 | 3.07E-09 |
| HELLPAR | 1.66E-09 |
| LINC02227 | 4.12E-14 |
| LCMT1-AS1 | 1.73E-22 |
| PRC1-AS1 | 8.73E-22 |
| ASB15-AS1 | 1.49E-09 |
| MMP25-AS1 | 8.09E-36 |
| LINC01239 | 4.15E-09 |
| LINC00159 | 2.79E-06 |
| CTBP1-AS | 2.28E-23 |
| ABCC5-AS1 | 6.46E-06 |
| LINC02709 | 8.78E-27 |
| LINC01985 | 3.32E-06 |
| LINC00824 | 1.24E-11 |
| RN7SL832P | 2.11E-24 |
| LINC01649 | 3.65E-30 |
| PRR29-AS1 | 2.55E-07 |
| CECR3 | 0.000150969 |
| LINC02805 | 1.01E-05 |
| LINC01772 | 1.42E-19 |
| LINC02528 | 1.82E-22 |
| GAS1RR | 2.28E-37 |
| ROCR | 6.73E-14 |
| LINC00632 | 1.35E-15 |
| LINC01250 | 7.28E-06 |
| KIF1C-AS1 | 1.76E-23 |
| MIR181A1HG | 7.86E-09 |
| WWTR1-IT1 | 6.69E-09 |
| LINC02033 | 8.91E-13 |
| LINC01989 | 8.37E-06 |
| CFLAR-AS1 | 7.19E-27 |
| RUSC1-AS1 | 1.31E-16 |
| LINC01819 | 4.09E-12 |
| PCA3 | 0.043089872 |
| RCCD1-AS1 | 4.05E-08 |
| BTG3-AS1 | 3.56E-32 |
| LINC02857 | 6.93E-33 |
| LINC02280 | 8.14E-27 |
| TEX26-AS1 | 9.55E-05 |
| CLDN10-AS1 | 4.78E-33 |
| IDH2-DT | 1.61E-38 |
| ARHGEF18-AS1 | 4.11E-07 |
| LINC01562 | 5.08E-06 |
| LINC01090 | 0.015711242 |
| SH3TC2-DT | 5.92E-08 |
| SPIN4-AS1 | 2.59E-06 |
| DLX6-AS1 | 4.60E-22 |
| LINC01094 | 6.62E-37 |
| LINC01252 | 6.12E-08 |
| NLGN1-AS1 | 3.51E-17 |
| DPYD-AS1 | 0.001028148 |
| LINC01841 | 1.04E-12 |
| LINC01612 | 2.61E-46 |
| PDC-AS1 | 1.38E-08 |
| LINC01524 | 0.003526185 |
| LINC00861 | 1.93E-31 |
| MRPS9-AS1 | 6.00E-12 |
| YEATS2-AS1 | 4.10E-30 |
| LINC01206 | 1.10E-09 |
| UFL1-AS1 | 2.91E-05 |
| LINC02453 | 1.74E-27 |
| VWA8-AS1 | 2.16E-33 |
| LINC00934 | 0.001612543 |
| DOCK4-AS1 | 1.36E-11 |
| PP12613 | 3.19E-23 |
| PLA2G4E-AS1 | 4.80E-14 |
| FLJ40194 | 1.05E-11 |
| LINC01970 | 0.01946585 |
| LRP4-AS1 | 1.81E-14 |
| LINC00885 | 1.68E-36 |
| PRICKLE2-AS2 | 0.00065213 |
| ATP11A-AS1 | 7.75E-16 |
| SCHLAP1 | 8.92E-52 |
| LINC01842 | 9.63E-13 |
| LINC02060 | 1.21E-06 |
| LINC01397 | 5.53E-08 |
| REV3L-IT1 | 0.024309171 |
| LINC00174 | 1.25E-17 |
| LNCARSR | 2.29E-06 |
| LINC02812 | 5.53E-09 |
| LINC01312 | 9.79E-29 |
| CAMTA2-AS1 | 7.45E-09 |
| F10-AS1 | 1.65E-27 |
| LINC00303 | 7.60E-14 |
| LINC00426 | 6.09E-28 |
| LINC02126 | 0.001353548 |
| GPC5-IT1 | 2.84E-33 |
| PAPOLA-DT | 4.06E-24 |
| PDXDC2P-NPIPB14P | 5.66E-16 |
| SAP30L-AS1 | 5.90E-11 |
| ZNF630-AS1 | 1.96E-05 |
| ZNF503-AS2 | 8.20E-35 |
| DUSP5-DT | 4.38E-08 |
| LINC01863 | 8.16E-27 |
| LINC01450 | 0.000140188 |
| LINC01585 | 5.16E-06 |
| ZNF32-AS2 | 8.30E-13 |
| ARHGAP27P1-BPTFP1-KPNA2P3 | 9.89E-23 |
| CACTIN-AS1 | 1.46E-14 |
| LINC02664 | 4.55E-22 |
| SNHG20 | 3.19E-25 |
| C20orf197 | 1.19E-32 |
| LINC02422 | 7.68E-13 |
| LINC00111 | 1.14E-15 |
| RNF216-IT1 | 1.61E-11 |
| ARHGAP26-IT1 | 4.23E-10 |
| LINC02362 | 8.55E-21 |
| LINC01224 | 2.36E-28 |
| LINC02803 | 1.45E-15 |
| BCAR4 | 2.25E-07 |
| LINC02278 | 7.35E-05 |
| TMEM78 | 1.39E-05 |
| ITPKB-IT1 | 0.000240687 |
| DLG1-AS1 | 7.54E-35 |
| SEC62-AS1 | 2.01E-08 |
| UBOX5-AS1 | 5.40E-16 |
| CASC11 | 9.84E-31 |
| MIR302CHG | 8.19E-08 |
| LINC02150 | 3.74E-12 |
| PCED1B-AS1 | 7.88E-36 |
| LMNB1-DT | 1.18E-05 |
| MIR3945HG | 4.21E-12 |
| LINC02552 | 5.82E-07 |
| WSPAR | 8.76E-59 |
| PGLS-DT | 4.93E-13 |
| RHOA-IT1 | 2.02E-12 |
| CXXC4-AS1 | 0.018655876 |
| CT70 | 0.000786497 |
| LINC01827 | 5.72E-18 |
| TMED2-DT | 4.88E-11 |
| LINC02388 | 0.02386028 |
| LINC02421 | 4.22E-18 |
| FAM13A-AS1 | 1.42E-29 |
| CARD11-AS1 | 2.18E-16 |
| PRR7-AS1 | 1.99E-33 |
| PRMT5-AS1 | 7.28E-09 |
| SEMA6A-AS1 | 1.72E-27 |
| LINC02614 | 8.01E-15 |
| PARP11-AS1 | 3.84E-05 |
| MPRIP-AS1 | 2.45E-05 |
| LINC01767 | 3.41E-19 |
| LINC01176 | 2.84E-21 |
| LINC02360 | 2.54E-12 |
| TRIM7-AS1 | 5.70E-13 |
| PLCG1-AS1 | 2.19E-22 |
| SMIM2-IT1 | 5.98E-05 |
| SPATA8 | 0.003299979 |
| LINC01934 | 6.79E-23 |
| LINC01888 | 7.74E-12 |
| CYLD-AS1 | 5.75E-29 |
| MRPL20-DT | 9.00E-22 |
| HMGA1P4 | 0.000448726 |
| FMNL1-DT | 4.20E-33 |
| LINC00347 | 0.0001187 |
| CSTF3-DT | 1.07E-08 |
| LINC00102 | 1.07E-09 |
| LIF-AS1 | 9.61E-09 |
| DNAH8-AS1 | 2.19E-11 |
| LINC02626 | 1.11E-09 |
| BEAN1-AS1 | 3.21E-13 |
| LINC02652 | 4.41E-09 |
| DNAH17-AS1 | 2.31E-07 |
| LINC02435 | 0.001935882 |
| LINC02416 | 6.71E-13 |
| EDRF1-AS1 | 2.91E-10 |
| LINC02751 | 5.61E-26 |
| ASAP1-IT2 | 8.05E-24 |
| SP2-DT | 2.72E-32 |
| LINC02225 | 4.88E-12 |
| LINC02832 | 0.010539358 |
| SLX1A-SULT1A3 | 3.46E-16 |
| ETV5-AS1 | 2.76E-07 |
| LINC02794 | 6.73E-05 |
| HNF4A-AS1 | 2.24E-20 |
| FAM215A | 1.44E-34 |
| LINC01030 | 1.03E-08 |
| SIRPG-AS1 | 5.62E-27 |
| GK-IT1 | 3.06E-09 |
| GNA14-AS1 | 0.004936546 |
| LINC00581 | 0.013707538 |
| NALCN-AS1 | 7.77E-06 |
| LINC02288 | 6.11E-09 |
| LINC02507 | 3.46E-06 |
| PITPNM2-AS1 | 1.24E-06 |
| PPM1K-DT | 1.21E-20 |
| LINC00603 | 2.02E-15 |
| MAP4K1-AS1 | 1.95E-16 |
| NPAS2-AS1 | 6.62E-05 |
| DTNB-AS1 | 1.75E-08 |
| MIR670HG | 4.85E-22 |
| LINC02067 | 1.60E-16 |
| LINC01999 | 0.002013878 |
| LINC00115 | 3.84E-10 |
| PDXP-DT | 3.00E-13 |
| LINC01344 | 0.001266396 |
| USP30-AS1 | 1.15E-28 |
| ATP2B1-AS1 | 8.78E-27 |
| SH3BP5-AS1 | 2.75E-13 |
| PTOV1-AS2 | 1.53E-18 |
| MIR202HG | 2.96E-06 |
| LINC01355 | 1.08E-20 |
| NCF4-AS1 | 1.55E-13 |
| LINC01987 | 9.68E-39 |
| LEMD1-DT | 0.000196418 |
| LINC01305 | 3.21E-10 |
| SPON1-AS1 | 1.65E-05 |
| NUCB1-AS1 | 1.13E-10 |
| SLC7A11-AS1 | 1.84E-11 |
| LINC01973 | 4.25E-09 |
| LINC02112 | 5.64E-05 |
| LINC01910 | 1.14E-09 |
| LINC01831 | 1.42E-46 |
| PRDM16-DT | 2.97E-40 |
| MIR155HG | 5.68E-33 |
| LINC02043 | 0.016828234 |
| DDR1-DT | 2.26E-05 |
| LINC00893 | 9.65E-17 |
| SSBP3-AS1 | 2.55E-10 |
| LINC01993 | 0.017690783 |
| MYCNUT | 8.48E-36 |
| LINC02481 | 5.79E-21 |
| CLEC12A-AS1 | 3.83E-22 |
| LINC01483 | 2.49E-15 |
| SLC24A3-AS1 | 0.002777276 |
| KIAA2012-AS1 | 1.53E-05 |
| EDIL3-DT | 1.53E-06 |
| CCL3-AS1 | 1.09E-33 |
