# Supplementary material for: Clinicopathological and Prognostic Value of Necroptosis-Associated lncRNA Model in Patients with Kidney Renal Clear Cell Carcinoma
Source: Dis Markers. 2022 May 23;2022:5204831. doi: 10.1155/2022/5204831 (PMC9157284; doi:10.1155/2022/5204831)
Supplement: Supplementary 4 — Supplementary Table S4: clinicopathological features of KIRC patients between the training and verification groups. [file 5204831.f4.docx]

Supplementary Table S4: Clinicopathological features of KIRC patients between the training and verification groups.

| Covariates | Total (%) | Training Cohort (%) | Verification Cohort (%) | p-value |
| --- | --- | --- | --- | --- |
| Age |  |  |  |  |
| <=65 | 347 (65.97%) | 173 (65.53%) | 174 (66.41%) | 0.903 |
| >65 | 179 (34.03%) | 91 (34.47%) | 88 (33.59%) |  |
| Gender |  |  |  |  |
| FEMALE | 183 (34.79%) | 93 (35.23%) | 90 (34.35%) | 0.905 |
| MALE | 343 (65.21%) | 171 (64.77%) | 172 (65.65%) |  |
| Grade |  |  |  |  |
| G1-2 | 239 (45.44%) | 120 (45.45%) | 119 (45.42%) | 1 |
| G3-4 | 279 (53.04%) | 140 (53.03%) | 139 (53.05%) |  |
| unknow | 8 (1.52%) | 4 (1.52%) | 4 (1.53%) |  |
| Stage |  |  |  |  |
| Stage I-II | 318 (60.46%) | 161 (60.98%) | 157 (59.92%) | 0.83 |
| Stage III-IV | 205 (38.97%) | 101 (38.26%) | 104 (39.69%) |  |
| unknow | 3 (0.57%) | 2 (0.76%) | 1 (0.38%) |  |
| T |  |  |  |  |
| T1-2 | 336 (63.88%) | 170 (64.39%) | 166 (63.36%) | 0.876 |
| T3-4 | 190 (36.12%) | 94 (35.61%) | 96 (36.64%) |  |
| M |  |  |  |  |
| M0 | 418 (79.47%) | 205 (77.65%) | 213 (81.3%) | 0.811 |
| M1 | 78 (14.83%) | 40 (15.15%) | 38 (14.5%) |  |
| unknow | 30 (5.7%) | 19 (7.2%) | 11 (4.2%) |  |
| N |  |  |  |  |
| N0 | 238 (45.25%) | 112 (42.42%) | 126 (48.09%) | 0.629 |
| N1 | 16 (3.04%) | 6 (2.27%) | 10 (3.82%) |  |
| unknow | 272 (51.71%) | 146 (55.3%) | 126 (48.09%) |  |

value
